# Influence of Typhoon Matsa on Phytoplankton Chlorophyll-*a* off East China

**DOI:** 10.1371/journal.pone.0137863

**Published:** 2015-09-25

**Authors:** Hui Zhao, Jinchao Shao, Guoqi Han, Dezhou Yang, Jianhai Lv

**Affiliations:** 1 Guangdong Key Lab of Climate, Resource and Environment in Continental Shelf Sea and Sea, College of Ocean and Meteorology, Guangdong Ocean University, Zhanjiang, China; 2 Northwest Atlantic Fisheries Centre, Fisheries and Oceans Canada, St. John’s, Canada; 3 Key Laboratory of Ocean Circulation and Waves, CAS, Qingdao, China; 4 Institute of Oceanology, Chinese Academy of Sciences, Qingdao, China; 5 Marine and Fishery Environment Monitoring Center of Guangzhou, Guangzhou, China; CAS, CHINA

## Abstract

Typhoons can cause strong disturbance, mixing, and upwelling in the upper layer of the oceans. Rich nutrients from the subsurface layer can be brought to the euphotic layer, which will induce the phytoplankton to breed and grow rapidly. In this paper, we investigate the impact of an intense and fast moving tropical storm, Typhoon Matsa, on phytoplankton chlorophyll-a (Chl-*a*) concentration off East China. By using satellite remote sensing data, we analyze the changes of Chl-*a* concentration, Sea Surface Temperature (SST) and wind speed in the pre- and post-typhoon periods. We also give a preliminary discussion on the different responses of the Chl-*a* concentration between nearshore and offshore waters. In nearshore/coastal regions where nutrients are generally rich, the Chl-*a* maximum occurs usually at the surface or at the layer close to the surface. And, in offshore tropical oligotrophic oceans, the subsurface maxima of Chl-*a* exist usually in the stratified water column. In an offshore area east of Taiwan, the Chl-*a* concentration rose gradually in about two weeks after the typhoon. However, in a coastal area north of Taiwan high Chl-*a* concentration decreased sharply before landfall, rebounded quickly to some degree after landfall, and restored gradually to the pre-typhoon level in about two weeks. The Chl-*a* concentration presented a negative correlation with the wind speed in the nearshore area during the typhoon, which is opposite to the response in the offshore waters. The phenomena may be attributable to onshore advection of low Chl-*a* water, coastal downwelling and intensified mixing, which together bring pre-typhoon surface Chl-*a* downward in the coastal area. In the offshore area, the typhoon may trigger increase of Chl*-a* concentration through uptake of nutrients by typhoon-induced upwelling and entrainment mixing.

## Introduction

When typhoons pass over the sea, they can effectively promote the marine phytoplankton, hence raising the sea surface Chl-*a* concentration. Chl-*a* concentration is an important index of marine photoautotroph, which is not only the primary producers of marine organic matter, but also the basic link in the marine food structure. It plays an important role in the process of material cycle and energy conversion in the marine ecological system, of relevance to marine atmospheric carbon cycle in the system, environment monitoring, ocean currents (upwelling and coastal current), fishery management and so on [1–7]. At present, many researchers who focus on the ocean, atmosphere and the environment believe that Chl-*a* concentration is an important parameter in the study of ocean color and marine ecological environment. Since global warming may bring stronger typhoons [8], their impact on marine phytoplankton Chl-*a* may be further enhanced.

The northwest Pacific and the East China Sea are frequently affected by typhoons. The influences of typhoons on the marine environments and climate are becoming a hot topic of research in the world. Each year, over the northwestern Pacific, 30 tropical cyclones on average are generated, roughly 7 of which make the landfall on China. Owing to the global warming and environment deterioration, the intensity of typhoons may increase [9]. The influences of typhoons on the marine environment, climate and ecosystem are drawing worldwide attention. Oceans get strong energy input from typhoons. Through upwelling, vertical mixing and entrainment, typhoons restructure stratification and bring rich nutrients from the subsurface layer to the euphotic layer, leading to a positive influence on the growth of phytoplankton. Accordingly the phytoplankton chlorophyll-a (Chl-*a*) concentration will also increase. Many studies have shown that typhoons play an important role in the Chl-*a* concentration increase [10–11].

In recent years, researchers studied influences of typhoons on the Chl-*a* concentration and primary productivity and roles of typhoons’ translation speed and forcing time in phytoplankton blooms. By using MODIS-Aqua ocean color remote sensing data, Lin et al. [12] investigated the phytoplankton bloom process caused by typhoon “Kai-Tak– 2000” in the South China Sea. The carbon fixation is estimated to be 0.8 Mt, about 2~4% of the primary productivity of the South China Sea in 2000. For typhoons, their net primary productivity contribution could be about 20~ 30% in the South China Sea. Zhao et al. [13] showed that through the study of two typhoons with different translation speed and maximum sustained speed, the influence of the fast moving typhoon was limited to the shallower upper ocean. However, the slowly moving one could cause quite strong phytoplankton blooms. Sun et al. [14] and Chen and Tang [15] also put forward that the important factor which leads to phytoplankton blooms is the enough time for forming a strong upwelling and inertial oscillation. Many studies showed that the reason that causes blooms in the offshore area is a series of physical processes, like upwelling, strengthened mixing and entrainment, which all increase sea surface nutrients. However, in the nearshore area, abundant rainfall and runoffs make a huge number of phytoplankton, colored dissolved organic matter and suspended sediment enter into the sea, which may lead to blooms [16–19]. In this study, we used satellite remote sensing data to analyze the changes of Sea Surface Temperature (SST), wind speed and the sea surface Chl*-a* concentration during Typhoon Matsa. We showed and discussed the different responses of sea surface Chl*-a* concentration between the nearshore and offshore areas.

## Data and Methods

### Satellite-derived data

Since typhoons have rapidly changing paths and intensities, setting mooring buoys or cruise survey costs enormously. It is hard to access to observational data comprehensively and systematically under the effect of typhoons. In such occasional and risky situations we can neither grasp the response in the upper ocean accurately nor get sufficient space coverage to estimate the phytoplankton blooms quantitatively caused by typhoons with traditional field surveys. So it’s difficult to thoroughly analyze the physical mechanism of the upper ocean response. With the development of technology, people overcome the constraints above by using satellite remote sensing. It provides an efficient method for getting comprehensive observational data in extreme weather events such as typhoons. Now, applying the technology based on satellite remote sensing is an important method to investigate the influence of typhoons on the ocean environment.

#### Sea surface temperature (SST)

The cloud-free daily SST images from TRMM Microwave Imager (TMI) onboard the Tropical Rainfall Measuring Mission (TRMM) is adopted. The data is available from December 7, 1997 (http://www.remss.com/missions/tmi). The spatial resolution of SST data is 0.25° × 0.25°, which was derived from Remote Sensing Systems sponsored by NASA’s Earth Science Research, Education and Applications Solution Network (REASoN) DISCOVER Project.

#### Typhoon track data

Track data is taken from the US Joint Typhoon Warning Center (JTWC) and Unisys (http://weather.unisys.com). It records the typhoon maximum sustained wind speed (MSW) and the location of typhoon center for every 6 hours, based on which the translational speed of the typhoon can be calculated.

#### QuikScat-derived sea-surface wind

The microwave scatterometer SeaWinds (QuikScat) data are employed to show the wind field of typhoon. QuikScat daily data is produced by Remote Sensing Systems, sponsored by the NASA Ocean Vector Winds Science Team (http://www.remss.com/missions/qscat). The spatial resolution is 0.25° ×0.25°.

#### MODIS-derived Chl-a concentration

**Fig 1:**
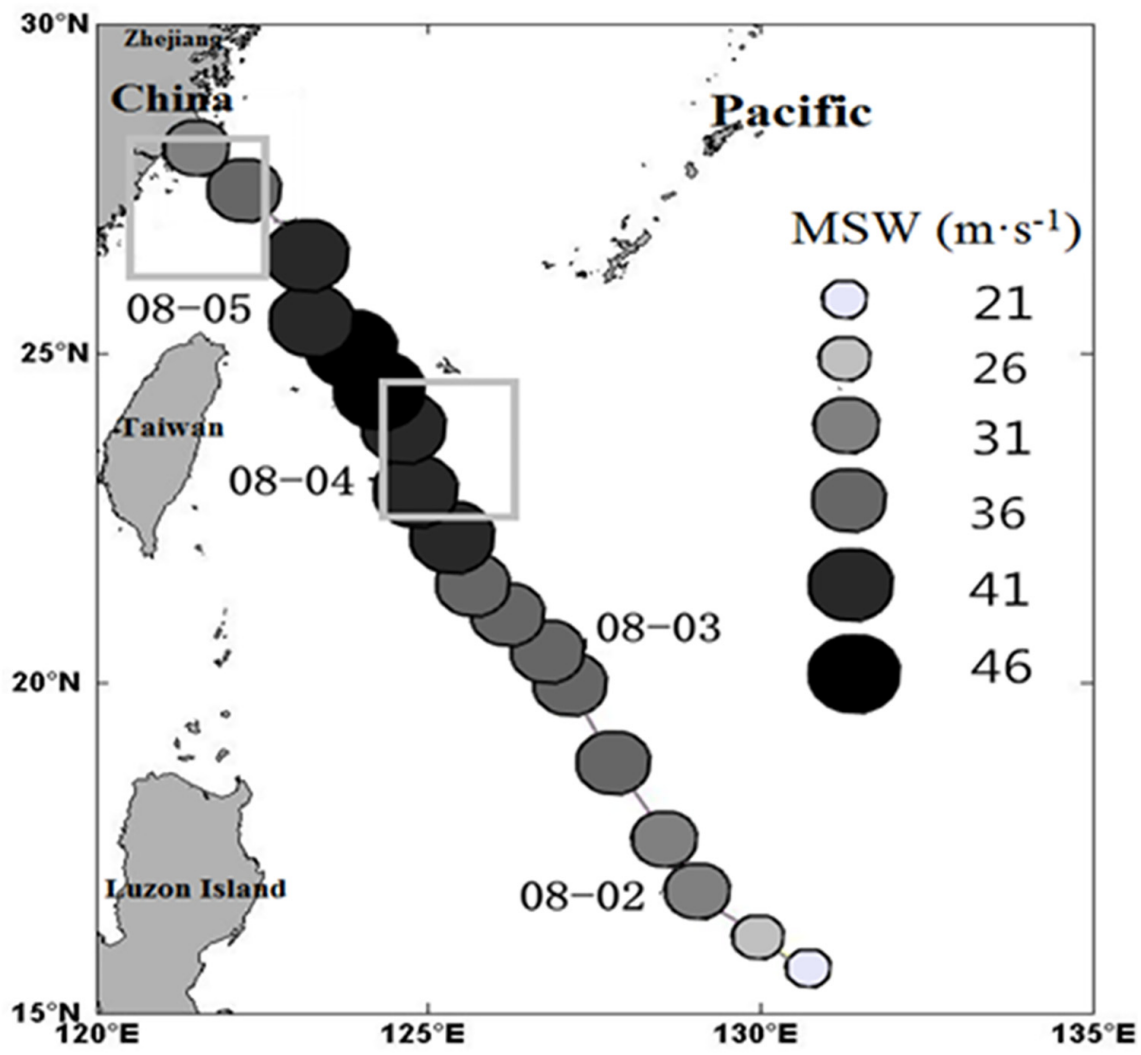
Map of the study domain and the two selected areas (nearshore: 26°N~28°N, 120.5°E~122.5°E, offshore: 22.5°N~24.5°N, 124.5°E~126.5°E). The track and intensity (the maximum sustained wind (MSW) speed) of Typhoon Matsa are also shown.

The L2 Chl*-a* concentration data are derived from MODIS-Aqua data (http://oceancolor.gsfc.nasa.gov/). We selected the area (15°N~30°N, 120°E~135°E) affected by Typhoon Matsa for mapping and analysis. Considering the typhoon path as well as the coverage of the Chl*-a* concentration data, we chose a box covering 26°N~28°N, 120.5°E~122.5°E as a representative of the nearshore area, and another box covering 22.5°N~24.5°N, 124.5°E~126.5°E as the representative of the offshore area (Fig 1). In this study, we analyze further the variations of SST, sea-surface wind and Chl*-a* concentration in these two areas.

### Computation of Ekman current and upwelling velocity

In order to investigate the possible mechanism of phytoplankton Chl*-a* response, the Surface Ekman current and Ekman pumping velocity in the nearshore area have been estimated from the QuikScat-derived sea-surface wind data (Box in Fig 1). We calculated the Ekman current at the surface as follows [20]:

$$V_{0}=\frac{0.0127}{\sqrt{\text{sin}|\varphi |}}U_{10}\;|\varphi |\geq 10,$$ (1)

Where *V*_0_ is the Surface Ekman current, *U*_10_ is the wind speed at 10 m above the sea and *φ* is the latitude. The direction of the surface Ekman Current is 45^o^ to the right of the wind direction in the northern hemisphere.

The Ekman pumping velocity (*W*_*E*_, positive upward) is calculated as

$$W_{E}\;=\;{curl}_{z}(\frac{\tau }{\rho_{W}f})$$ (2)

Where z is the vertical coordinate positive upward, *ρ*_*w*_ is the water density, *f* is the Coriolis parameter, and *τ* is the wind stress on the ocean surface calculated as

$$\tau \;=\;\rho_{a}C_{D}|U_{10}|U_{10}$$ (3)

Where *ρ*_*a*_ is the air density and *C*_*D*_ is the drag coefficient.

The coastal upwelling velocity (*W*_*c*_, positive upward) associated with the upwelling-favorable alongshore wind is calculated as [21]

$$W_{c}\;=\;\frac{\tau_{a}}{\rho_{W}fL}$$ (4)

Where *τ*_*a*_ is the alongshore wind stress and *L* is the horizontal length scale of upwelling.

## Results

### The path of typhoon Matsa and wind field

Typhoon Matsa originated at 10.5°N, 136.0°E on July 31, 2005 as a tropical depression, and became a strong tropical storm east of Philippines on August 1. On August 2, it was strengthened into a category 1 typhoon northeast of Luzon island and intensified into a Category 2 typhoon east of Taiwan Island on August 4. Matsa landed in southeast Zhejiang, China on August 5. The typhoon data provided by NOAA Climate Prediction Center, including the wind speed, translation speed and location of the typhoon centre are shown in Table 1.

**Table 1:** Positions, times, intensities and translation speeds of Typhoon Matsa.

Lat (°N)	Lon (°E)	Time	MSW (m·s-1)	TS (m·s-1)
10.4	135.5	07/31/03Z	13	7.3
11.3	134.4	07/31/09Z	18	6.6
11.7	133.9	07/31/12Z	18	8.8
13.1	132.9	07/31/18Z	21	3.9
13.8	132.6	08/01/00Z	23	7.4
15.0	131.8	08/01/06Z	23	6.7
15.7	130.7	08/01/12Z	23	4.1
16.1	130.0	08/01/18Z	28	5.9
16.8	129.1	08/02/00Z	33	6.1
17.8	128.5	08/02/06Z	33	6.3
18.8	127.8	08/02/12Z	36	7.1
20.0	127.1	08/02/18Z	39	4.0
20.6	126.6	08/03/00Z	39	4.4
21.2	126.0	08/03/06Z	39	3.3
21.7	125.6	08/03/12Z	39	3.3
22.3	125.4	08/03/18Z	41	5.1
23.1	124.8	08/04/00Z	41	4.7
24.0	124.7	08/04/06Z	41	3.7
24.6	124.3	08/04/12Z	46	5.1
25.3	123.6	08/04/18Z	46	2.2
25.6	123.3	08/05/00Z	44	5.2
26.6	123.2	08/05/06Z	41	6.9
27.5	122.2	08/05/12Z	39	5.1
28.2	121.5	08/05/18Z	33	3.3

MSW: Maximum wind speed; TS: Translation speed.

**Fig 2:**
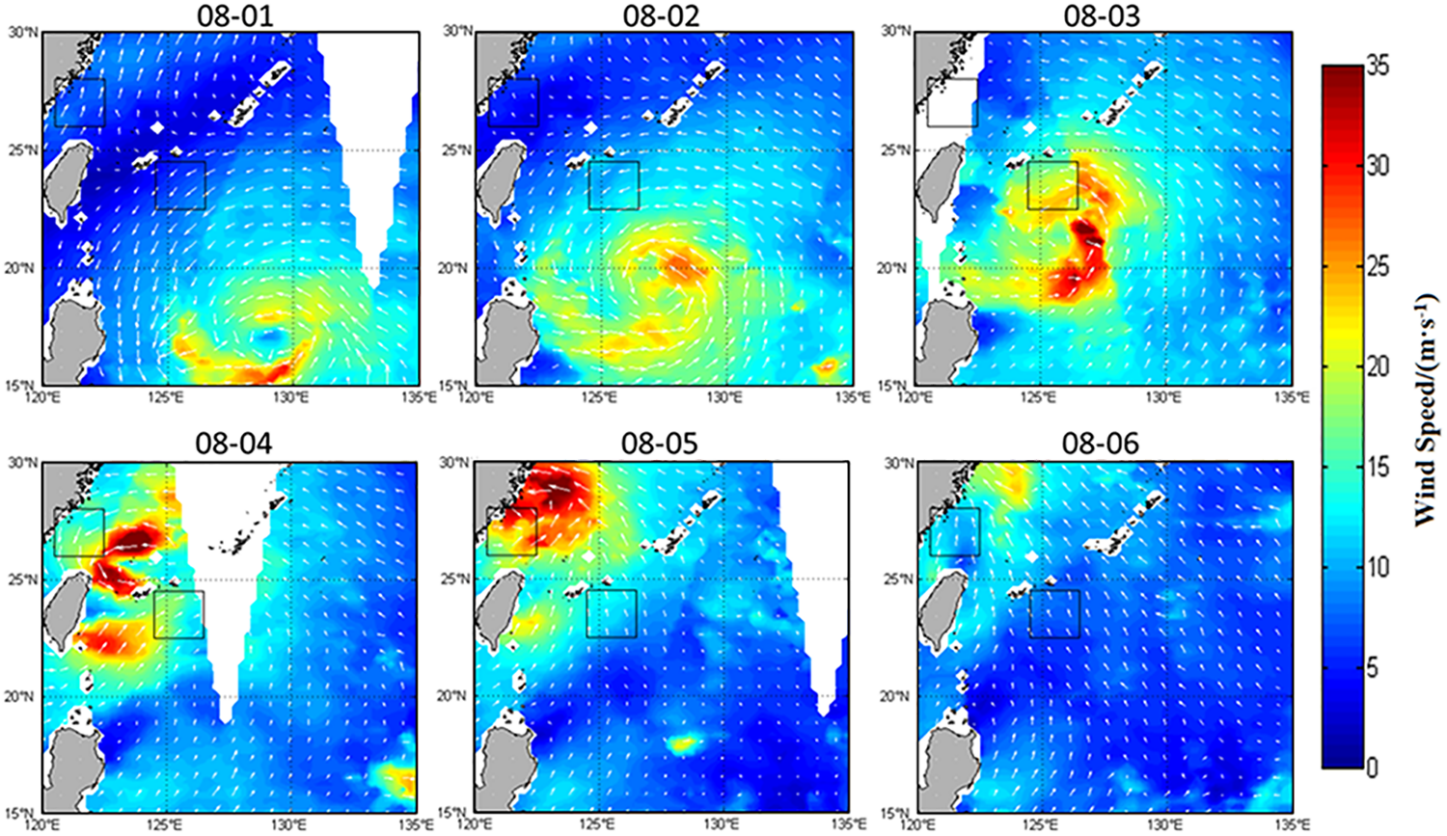
Sea-surface wind from August 1 to 6, 2005 (arrows represent the wind directions).

**Fig 3:**
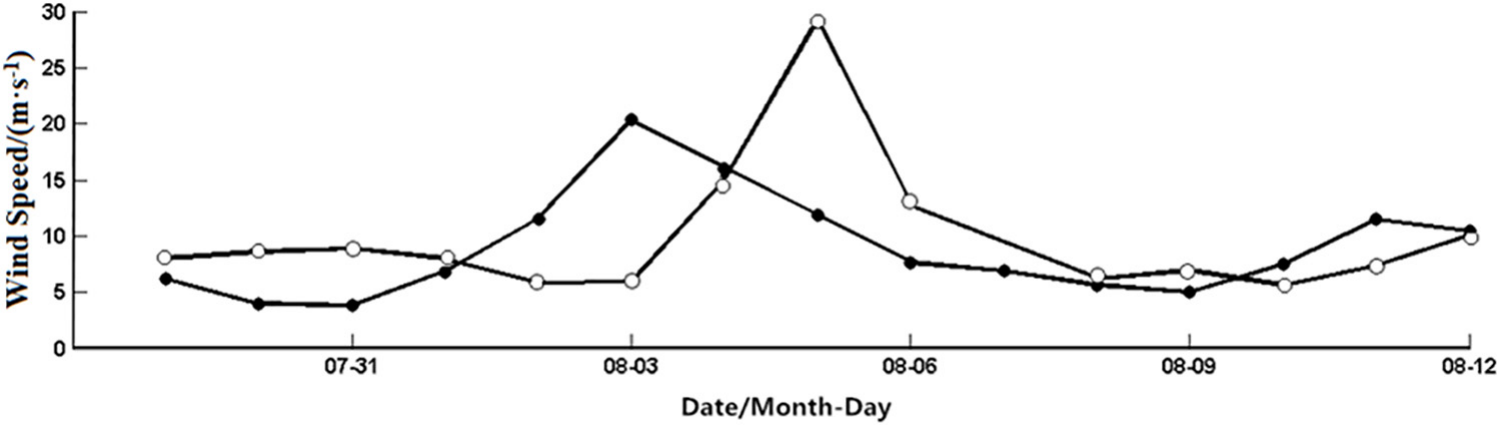
Time series of sea-surface winds spatially averaged for the nearshore (open dots) and offshore (solid dots) boxes from July 29 to August 12, respectively.

The structure of typhoon and ambient environments determine its northwestward movement in the northwestern Pacific [22–24]. Matsa enhanced gradually as it travelled to the northwest, with the maximum sustained wind speed up to about 50 m·s^-1^. Sea surface winds (Figs 2 and 3) show significant changes in the nearshore and offshore boxes during the typhoon. The time series (Fig 3) indicates that the typhoon forcing time (Fig 3) was from August 2 to 5 in the offshore box, with a maximum wind speed of about 20 m·s^-1^ on August 3. Matsa impacted the nearshore box from August 3 to 6, with the maximum wind speed of about 29 m·s^-1^ on August 5. The wind speed in both the nearshore and offshore boxes was quickly restored to the pre-typhoon level about 3 days after the typhoon passage.

### The influence of Typhoon Matsa on SST

One of the most significant ocean responses to a typhoon is the decrease of SST [25]. SST also plays a key role in the emergence, evolution and intensification of a typhoon. The fall of SST is influenced by the typhoon strength, speed and other factors. On the other hand, the upper ocean structure itself and local marine background environment around the typhoon also affect SST. Owing to the low pressure and intense wind stress, typhoon causes strong mixing and divergence in the upper ocean. Entrainment induced by typhoon makes the mixed layer deepen, and upwelling causes shoaling of the thermocline and a regional bulge in the nitracline [26], which results in the SST fall. The SST fall can reduce the heat flux transported from the ocean to the atmosphere, and then weaken the typhoon, forming a negative feedback process gradually [27]. As a result, the typhoon process is one of the most complicated and strongest air-sea interactions. Typhoon brings intense mixing to the upper ocean and a large amount of nutrients to the ocean surface at the same time, which promotes the growth of phytoplankton to form a large area of algal blooms. It increases marine primary productivity and has extremely important effect on marine ecosystems.

**Fig 4:**
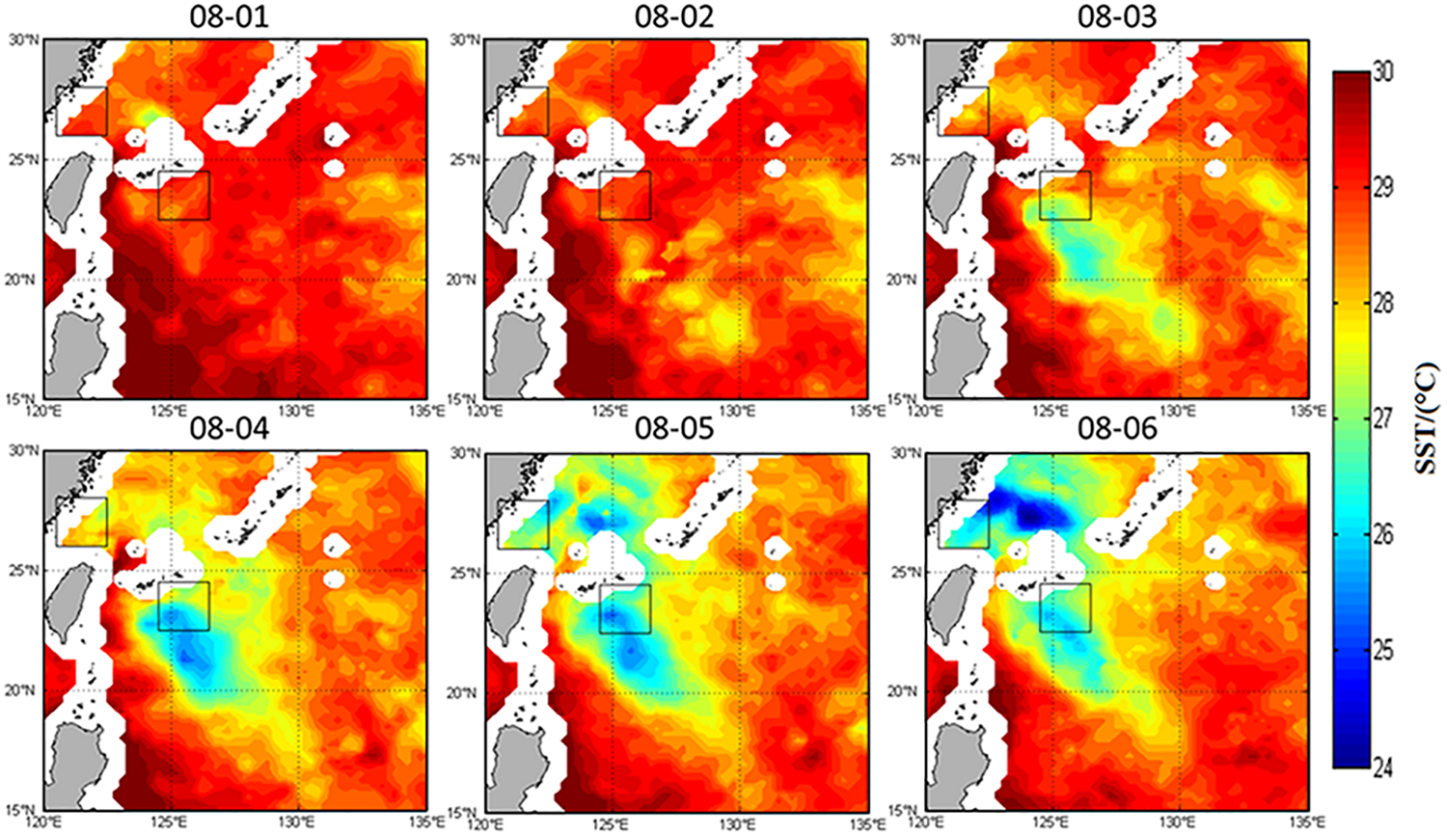
SST from August 1 to 6, 2005.

**Fig 5:**
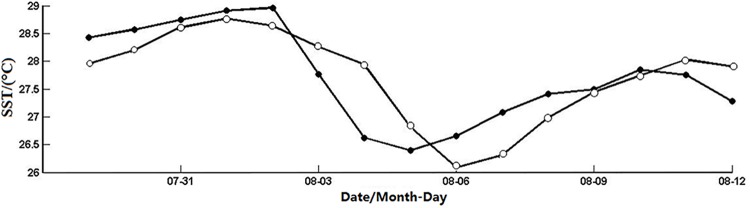
SST in the nearshore (open dots) and offshore (solid dots) boxes from July 29 to August 12, 2005.

Fig 4 indicates significant impacts of typhoon Matsa on SST both nearshore and offshore. The most pronounced SST decline was near typhoon’s path. SST (Figs 4 and 5) began to decline around August 2, between 19°N~24°N. As Matsa moved to the northwest, it enhanced gradually and finally reached the maximum intensity in 25°N~30°N from August 4–6,where the typhoon moved relatively slowly and SST decreased significantly. The regional average of SST (Fig 5) in the offshore box decreased by 9% from 29°C on August 2 to 26.4°C on August 5. It recovered to the peak on August 10 after typhoon. Thus, the SST responses were very obvious in both the nearshore and offshore areas near the typhoon path.

### The influence of Typhoon Matsa on Chl-*a*

**Fig 6:**
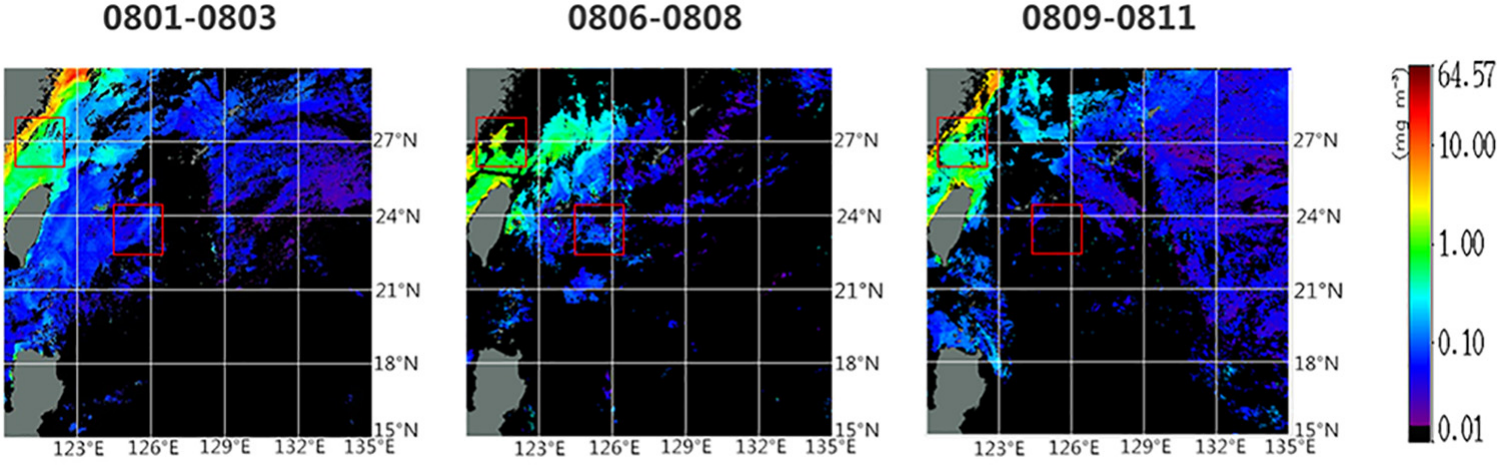
Sea surface Chl*-a* concentration during the pre- and post-typhoon periods.

The Chl*-a* concentration inversion channel based on satellite is in the visible range, which can hardly penetrate the cloud. So the Chl*-a* data may be severely corrupted by the cloud during typhoon, especially in the typhoon center. It’s difficult to analyze from the image in a single day, where the data loss is severe. Therefore, merging data for days is necessary. Here, Chl*-a* concentration data are averaged for consecutive 3 days (i.e. August 1 to 3, August 6 to 8, August 9 to 11, respectively), showing the changes between pre- and post-typhoon (Fig 6).

Low Chl*-a* concentration (Fig 6) was observed in most of the open sea. There are high pre-typhoon Chl*-a* values in coastal regions off Zhejiang, which may be caused by the summer-monsoon-induced coastal upwelling, shallow depth and the possible release of terrestrial materials [28].

**Fig 7:**
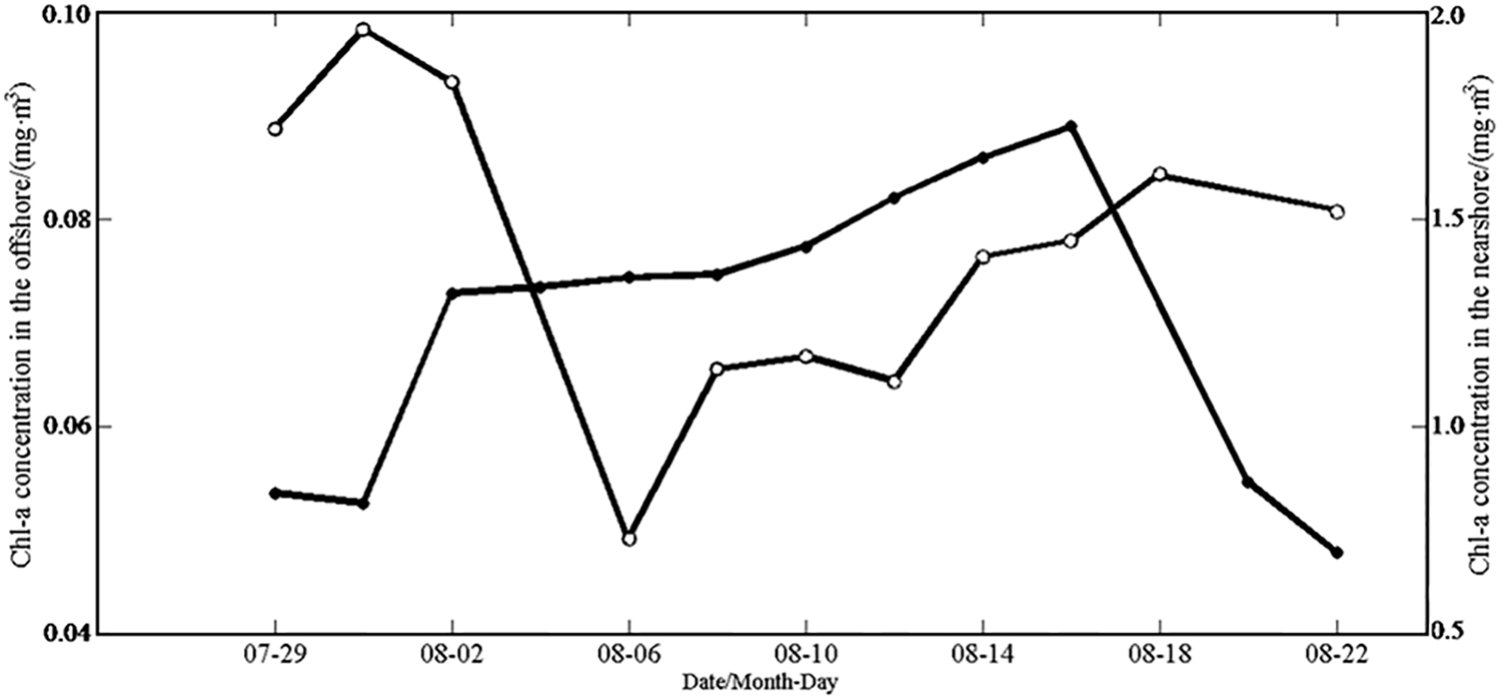
Sea surface Chl*-a* concentrations in the nearshore area (open dots) and the offshore area (solid dots). Chl*-a* data are not available for the offshore area on 4 August or 16 August, or in the offshore area on 20 August, 2005.

The time series of 48-h averaged Chl-*a* concentrations (Fig 7) were also shown for the nearshore box. As the typhoon gradually approached the nearshore region on August 4–5, the data missing became more severe in that region, and we did not include these two days’ data. The nearshore Chl*-a* concentration dropped obviously around August 6, from 1.83 to 0.73 mg·m^-3^ (by 60.1%); When the typhoon moved away and wind weakened around August 8, the Chl*-a* concentration restored to 1.17 mg·m^-3^, an increase of 60.3%. The Chl*-a* restored roughly back to the pre-typhoon level two weeks after (i.e. 18 August, 2005).

The time series of Chl*-a* concentrations (Fig 7) indicated that though the Chl*-a* concentration was low in the offshore box, it kept increasing for a longer time after the typhoon, reaching a maximum of 0.09 mg·m^-3^ on August 17 (increasing by 78% over the pre-typhoon value). Then it began to decline gradually, to the pre-typhoon level on August 23.

**Fig 8:**
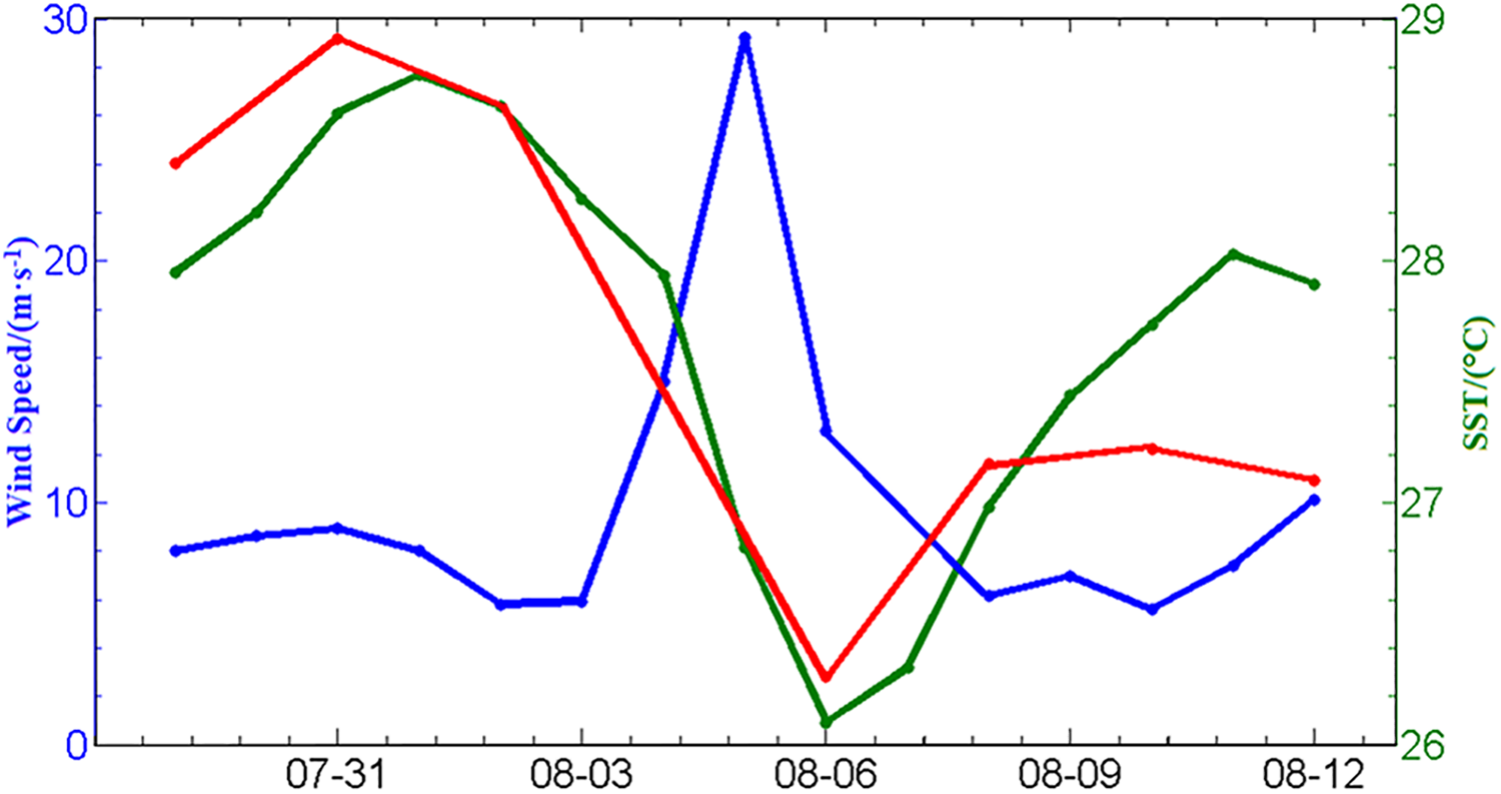
SST, Chl*-a* concentration and wind speed in the nearshore area during the typhoon.

The changes the Chl*-a* concentration and SST during Typhoon Matsa appear to be closely related to the wind speed in the nearshore box (Fig 8). The SST and Chl*-a* concentration decreased as the wind speed increased.

## Discussion

### Responses of Chl-*a* in the offshore region

**Fig 9:**
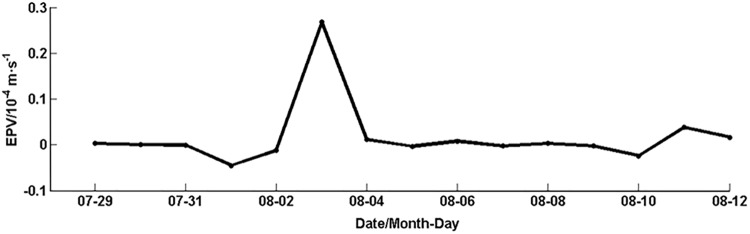
Ekman pumping velocity (EPV, positive upward) in the offshore area during the typhoon.

**Fig 10:**
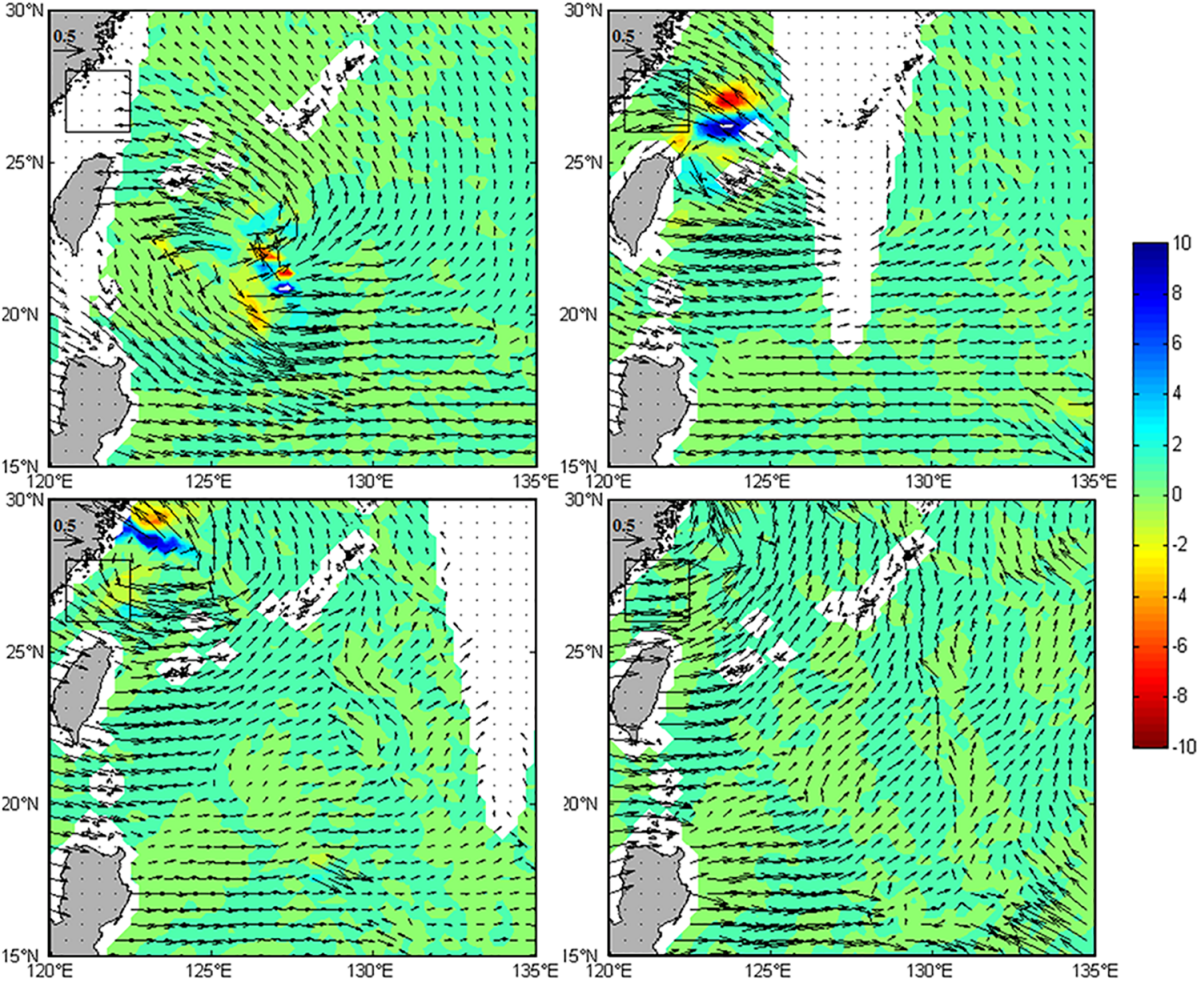
Surface Ekman current and Ekman pumping velocity (EPV) from August 3~6, 2005. Surface Ekman current vectors (arrows represent current speeds in m·s^–1^ and their directions) and EPV (color shaded in 10^−4^ m·s^–1^).

Light conditions and nutrients are two main ecological factors controlling the phytoplankton Chl*-a* concentration in the upper oceans [29–30]. Their spatial structures generally determine the vertical distribution of Chl*-a* concentration [31]. A bell-shaped vertical profile of Chl*-a* concentration, conventionally referred to as a subsurface chlorophyll maximum (SCM) phenomenon, has frequently been observed in stratified oceans. Therefore Chl-*a* maximum value generally appears in subsurface layer with relatively rich nutrients in the stratified water columns of offshore deep oceans. The subsurface maxima of chlorophyll [32] are usually produced in certain regions of the water column where two opposing resource (light and nutrient) gradients combined with turbulent mixing are amenable for survival of phytoplankton. Chen et al. [33] affirmed also that existence of summer SCM in the offshore region east of Taiwan with a bell-shaped vertical profile of Chl*-a* concentrations, where the maximum Chl*-a* layer is near the depth of 100 m. In the present study, the response of the Chl*-a* concentration to typhoons in the offshore area is consistent with previous research [19]. Strong wind brought by Matsa caused the strong disturbance, mixing and upwelling. The Ekman pumping velocity (Figs 9 and 10) enhanced significantly during the typhoon passage. These processes could bring nutrients/high phytoplankton concentrations from the subsurface water to the euphotic layer, increasing sea surface phytoplankton Chl*-a*. In view of the lag time of phytoplankton growth for uptake of nutrients (the average turnover time being 2–6 days [1]), the slower increase of the Chl*-a* after the typhoon in the offshore region may also suggest possibility of nutrients into the surface layer.

### Responses of Chl*-a* concentrations in the nearshore region

**Fig 11:**
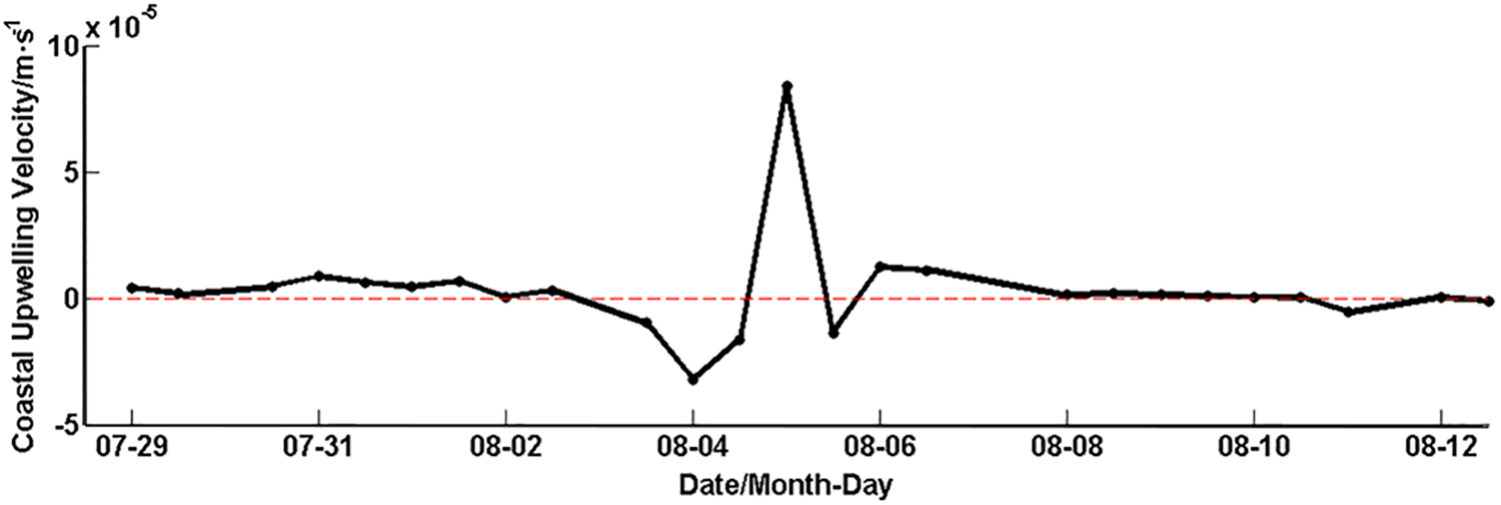
Coastal upwelling velocity for the nearshore box (positive upward), derived from Eq (4) by assuming a cross-shelf scale of 200 km. .

On the contrary, the Chl*-a* concentration responded to the typhoon rapidly in the nearshore region, decreasing to the minimum 1 day after the typhoon passage and recovering to 1.17 mg·m^-3^, with an increase over 60% of the minimum 2~3 days later. Previous studies [32,34–36] indicate that nutrients were generally rich in nearshore/coastal regions with enough sunlight, where the chlorophyll maximum occurs usually at the surface or at the layer close to the surface, and then the Chl*-a* concentration decreases with depth. The vertical mixing caused by the strong wind of typhoon likely reduced the surface high Chl*-a* concentration. This phenomenon was also reported by Zhou et al. [37] in Hong Kong waters. On the other hand, the surface Ekman flow can advect low Chl*-a* oceanic water onshore (Fig 10), causing the coastal downwelling (Fig 11, the downwelling velocity is 3.2×10^−5^ m·s^-1^ on August 4) and thus the increase of the subsurface Chl*-a* concentration, as well as the surface Chl-*a* decrease during the typhoon’s approach to the coastline. It is likely that this process created a transient SCM in the nearshore area. However, this downwelling process was soon replaced by an upwelling process (Fig 11, the upwelling velocity is 8.4×10^−5^ m·s^-1^ on August 5) after landfall, such that the Chl*-a* in the transient SCM was brought up to the surface, leading to the rapid, partial rebound of the surface Chl*-a* concentration. In the meantime, the upwelling process also caused the re-suspension of rich nutrients, which led to the eventual restoration of the Chl*-a* concentration to the pre-typhoon level. Therefore, the pattern of Chl*-a* in the nearshore area during the typhoon may mainly be regulated by typhoon-induced mixing, onshore advection, and coastal downwelling and upwelling.

### Conclusions

Satellite remote sensing data were used to investigate the responses of Chl*-a* to physical disturbances induced by Typhoon Matsa in the East China Sea. We arrived at the following conclusions: (1) SST significantly dropped in both the nearshore and offshore areas after the typhoon passage. (2) In the nearshore area, the Chl*-a* concentration decreased sharply in a couple of days after the typhoon passage, recovered to some degree soon after, and restored to the pre-typhoon level in about 2 weeks. In the offshore area the Chl*-a* concentration increased gradually in about 2 weeks after typhoon. (3) In the nearshore area, the rapid responses (decrease) of Chl*-a* were probably attributable to the direct wind mixing and the coastal downwelling associated with the onshore surface Ekman transport which advects low Chl*-a* water towards the coast. The coastal downwelling was replaced by upwelling after the typhoon landfall, resulting in a rapid partial restoration of the Chl*-a* concentration, through the uptake of the sub-surface Chl*-a* which was likely enhanced by the mixing and downwelling before landfall.
